# European Society of Organ Transplantation Consensus Statement on Testing for Non-Invasive Diagnosis of Kidney Allograft Rejection

**DOI:** 10.3389/ti.2023.12115

**Published:** 2024-01-04

**Authors:** Sookhyeon Park, Joana Sellares, Claire Tinel, Dany Anglicheau, Oriol Bestard, John J. Friedewald

**Affiliations:** ^1^ Feinberg School of Medicine, Northwestern University, Chicago, IL, United States; ^2^ Vall d’Hebron University Hospital, Barcelona, Spain; ^3^ Dijon University Hospital, INSERM UMR 1098 Right, UBFC, Dijon, France; ^4^ Necker Hospital, Assistance Publique-Hopitaux de Paris, INSERM U1151, Université Paris-Cité, Paris, France

**Keywords:** biomarkers, kidney transplant, rejection, non-invasive, diagnostics, gene expression, cell-free DNA, urine chemokines

## Abstract

To address the need for improved biomarkers for kidney transplant rejection, European Society of Organ Transplantation (ESOT) convened a dedicated working group comprised of experts in kidney transplant biomarkers to review literature pertaining to clinical and subclinical acute rejection to develop guidelines in the screening and diagnosis of acute rejection that were subsequently discussed and voted on during the Consensus Conference that took place in person in Prague. The findings and recommendations of the Working Group on Molecular Biomarkers of Kidney Transplant Rejection are presented in this article.

## Introduction

The short- and long-term success of kidney transplants relies on the safe and effective prevention of allograft rejection. Monitoring the alloimmune response to the kidney graft has been done for decades by serial measurements of graft function (non-invasive measuring of serum creatinine) and immunosuppressive drug levels and employing both reactive “for-cause” and systematic “surveillance” allograft biopsies. Monitoring serum creatinine has been demonstrated to be an insensitive and lagging indicator of allograft rejection and injury [1–4] and immunosuppressive drug level monitoring may inform efficacy for groups of patients but is not suited to individual rejection monitoring outside the extremes [5]. Thus, there is a significant unmet need for a more sensitive and specific non-invasive monitoring tool for allograft rejection and the adequacy of immunosuppression that can reduce or eliminate the need for surveillance biopsies and inform the need for indicated biopsies.

The target population for improved non-invasive tests for rejection would include all patients with a functioning kidney transplant. While rates of clinical and subclinical rejection are highest in the first 2 years post-transplant, possibly due to detection bias, patients are always at risk of alloimmune graft injury if they are functionally under-immunosuppressed, regardless of the cause. The non-invasive biomarkers addressed in this review have been introduced into clinical practice around the world in various combinations and at various times throughout the transplant patient journey. The goal of this review is to provide a snapshot of the current published evidence for their use and to provide a roadmap for the future development and implementation of these technologies.

To address the need for evidence-based guidelines for the adoption of molecular biomarkers in kidney transplantation, ESOT convened a consensus conference, comprised of a global panel of experts to develop guidelines on key aspects of non-invasive biomarkers of rejection. Summaries of the evidence were presented to the entire group of panelists and juries. The consensus findings and recommendations of the ESOT Consensus guidelines on molecular biology testing for non-invasive diagnosis of kidney allograft rejection are presented in this document. This document, which will be updated to reflect new evidence as it becomes available, is intended for healthcare providers.

## Methods

The consensus development process was organized by a dedicated Guidelines Taskforce within ESOT and its sections ELITA, EKITA, EPITA, ECTTA, ETHAP, Education Committee, YPT, Transplant International editorial board members and patient representatives. The detailed description of the methodology used is reported previously [6].

Briefly, key issues related to the topic of non-invasive biomarkers for kidney transplant rejection were identified by each working group and specific clinical questions were formulated according to the PICO methodology (PICO = Population, Intervention, Comparator and Outcome). All PICO questions are listed in subsequent sections of the manuscript. Following the definition of the PICOs, literature searches were developed by expert staff from the CET who have expertise in conducting systematic reviews and subsequently integrated, when needed, by the steering committee experts.

**FIGURE 1:**
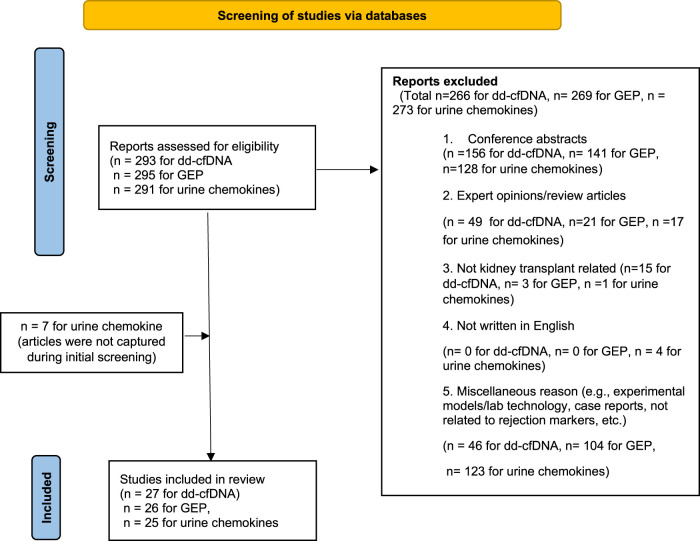
Flowchart summarizing the selection process of studies included in the evaluation of biomarkers for rejection in kidney transplantation. *Modified figure from:* [38]. For more information, visit: http://www.prisma-statement.org/.

A PRISMA flowchart describing the number of studies identified by the literature search and the number of studies selected for inclusion in the consensus statement appears in Figure 1.

A summary of the key evidence addressing each key question by the included studies was prepared in evidence Tables 1–5. The primary studies are included in these tables. Additional studies reviewed but not included in the manuscript are included in Supplementary Appendix SA. The workgroup proposed a recommendation for each key question based on the quality of evidence rated using the GRADE approach, with high quality rated as A, medium quality as B, and low quality as C; very low quality of evidence was not considered. For evaluation of the quality of evidence according to GRADE [33], the following features were considered: study design, risk of bias, inconsistency, indirectness, imprecision, number of patients, effect, importance, and publication bias. The strength of recommendation was rated as 1 (strong) or 2 (weak).

**TABLE 1:** Summary of key literature reviewed on donor-derived cell-free DNA for subclinical rejection.

Authors	Study design	Number of samples	Results
[2]	Retrospective analysis on biorepository samples, Single center	114 biopsies	For any subAR (AUROC 0.89, PPV 0.55, NPV 0.97)
		13 rejections	
		101 no rejection	
[7]	Prospective observational (*post hoc* analysis), Multicenter	428 biopsies	For any subAR (AUROC 0.72, PPV 0.56, NPV 0.84)
		103 rejections	
		325 no rejection	
[8]	Retrospective cross-sectional, Single center	37 biopsies	For any subAR (sensitivity 0, specificity 0.89, PPV 0, NPV 0.71)
		10 rejections	
		27 no rejection	

AUROC, area under the receiver operating characteristic curve; subAR, subclinical rejection; NPV, negative predictive value; PPV, positive predictive value.

**TABLE 2:** Summary of key literature reviewed on donor-derived cell-free DNA for clinical acute rejection.

Authors	Study design	Number of samples	Results
[9]	Prospective observational, Multicenter	107 biopsies	For any rejection (AUROC 0.74, PPV 0.61, NPV 0.84)
		27 rejections	
		80 no rejection	
[10]	Subgroup analysis of prospective observational, Multicenter	87 patients	For ABMR with + DSA and dd-cfDNA >1%, (AUROC 0.86, PPV 0.81, NPV 0.83]
		16 ABMR	
		53 no rejection	
[2]	Retrospective analysis of biorepository samples, Single center	217 biopsies	For any rejection for cause + SubAR (AUROC 0.87, PPV 0.52, NPV 0.95)
		38 rejections	
		179 no rejection	
[11]	Prospective observational, Single center	63 biopsies	For any rejection (AUROC 0.71, PPV 0.77, NPV 0.75)
		34 rejections	
		29 no rejection	
[12]	Prospective observational, Single center	189 patients	For any rejection (Absolute concentration of dd-cfDNA (AUROC 0.83) is better than dd-cfDNA (%) (AUROC 0.73)
		22 rejections	
		395 stable samples	
[13]	Prospective cross-sectional, Multicenter (*n* = 2)	61 biopsies	For ABMR (absolute concentration AUROC 0.91 vs. dd-cfDNA (%) 0.89)
		13 ABMR	
		48 no rejection	
[14]	Subgroup analysis of prospective observational, Multicenter	79 patients with TCMR 1A/borderline changes	Subjects with TCMR (1A and borderline) with high dd-cfDNA had worse clinical outcomes compared to those with low dd-cfDNA
[15]	Cross-sectional for DSA screening/Retrospective testing of dd-cfDNA on bio-banked samples, Single center	From 2 independent cohort	For ABMR with +DSA
		45/30 biopsies	AUROC for dd-cfDNA alone 0.89/0.69
		25/17 ABMR	AUROC for DSA alone 0.88/0.77
		20/13 no ABMR	
[3]	Prospective observational, multicenter (ADMIRAL)	219 biopsies	For any rejection dd-cfDNA (AUROC 0.8, PPV 0.5, NPV 0.9)
		113 rejections	
		106 no rejection	
[16]	Prospective observational, Single center	208 biopsies	For any rejection dd-cfDNA and MMDx (AUROC 0.80), dd-cfDNA and histology (AUROC 0.75)
		162 rejections by histology	
		46 no rejection by histology	
[17]	Prospective observational, multicenter (TRIFECTA)	300 biopsies	dd-cfDNA levels are strongly associated with the active molecular rejection phenotype (MMDx), particularly with AMR, mixed, and active TCMR
		120 rejections	
		180 no rejection	
[18]	Prospective observational, multicenter (TRIFECTA)	367 biopsies	Any rejection prediction AUROC in test set by logistic regression model using both dd-cfDNA (%) and absolute concentration
		83 (histology test set) rejection	• 0.88 for MMDx
		71 (MMDx test set) rejection	• 0.82 for histologic rejection

AUROC, area under the receiver operating characteristic curve; DSA, donor-specific antibody; MMDx, the molecular microscope diagnostic system; NPV, negative predictive value; PPV, positive predictive value.

**TABLE 3:** Summary of literature review on GEP for clinical and subclinical acute rejection.

Authors	Study design	Biomarker	Number of samples	Results
[4]	Multicenter; multiple biorepository retrospective validation sets	17 gene signatures	237	For subAR (including BL) vs. No rejection. AUROC 0.83, NPV 0.89; PPV 0.73
		RNA seq (Tuteva)	46 subAR	For AR (including BL) sets vs. No rejection AUROC 0.81–0.97 (in biorepository validation sets)
			145 No rejection	
[1]	Multicenter prospective, internal validation for discovery and validation sets	57 gene signature Microarray/qPCR (120 genes) (TruGraf)	382 143 subAR 239 stable	For subAR (including BL) vs. Stable. AUROC 0.85 NPV 0.88/PPV 0.61
[7]	Post-hoc analysis from a prospective observational	Combined	408	For subAR (including BL) vs. Stable AUROC 0.75, NPV 0.82, PPV 0.47
		TruGraf + dd-cfDNA	103 subAR	
			325 stable	
[19]	Multicenter with external retrospective sample validation	23 gene signature RNA seq (Clarava)	155	For AR (mostly subAR + cAR including BL) vs. No rejection AUROC 0.74, NPV 0.88, PPV 0.70
			For discovery set: 32 AR (cAR + subAR)	
			49 no rejection	
[20]	Multicenter prospective, internal validation for discovery and validation sets	13 12-gene signature/RT-PCR fluidigm (kSORT)	558	For AR (subAR and cAR including BL) vs. No rejection AUROC 0.95; Sen 0.92, Spec 0.93
			187 AR (cAR + subAR)	
			371 No rejection	
[21]	Multicenter validation cohort	17-gene rt-PCR (kSORT)	1763	For AR (cAR +subAR including BL) vs. No rejection AUROC 0.51
			188 AR (cAR +subAR)	
			1575 No rejection	
[22]	Multicenter retrospective, internal validation for discovery and validation sets	5-gene signature RT-PCR/RNAseq (Allomap kidney)	191	For AR (cAR +subAR) vs. stable AUROC 0.78
			47 AR (cAR +subAR)	NPV 0.9–0.95, PPV 0.23–0.48
			146 stable	
[23]	Multicenter, prospective validation cohort	5-gene signature RT-PCR/RNA seq (Allomap kidney)	235	For AR (clinical and subclinical) vs. stable Sen 0.7, Spec 0.66 NPV 0.95
			66 AR	
			169 stable	
[24]	Multicenter prospective, internal validation for discovery and validation sets	8-gene signature	384	For ABMR (cAR +subAR) vs. no ABMR AUROC 0.80 NPV 0.96, PPV 0.26
		RT-PCR/RNAseq	186 ABMR (cAR +subAR)	
			248 no ABMR	

ABMR, antibody-mediated rejection; AR, acute rejection; AUROC, area under the receiver operating characteristic curve; BL, borderline; cAR, clinical acute rejection; subAR, subclinical acute rejection; NPV, negative predictive value; PPV, positive predictive value; Sen, sensitivity; Spec, specificity.

**TABLE 4:** Summary of key literature reviewed on urine chemokines for subclinical acute rejection.

Authors	Study design	Number of samples	Results
[25]	Retrospective analysis, CXCL10, Single center	102 biopsies	For scTCMR (including BL) versus normal (AUROC 0.85; OR 1.41)
		30 subAR	
		22 normal	
[26]	Retrospective analysis, CXCL10, Single center	362 biopsies	For subAR (including BL) versus no rejection (AUROC 0.69)
		119 subAR	
		243 no rejection	
[27]	Prospective longitudinal analysis, CXCL9 and CXCL10, Single center	1722 samples	For subAR (excluding BL)
		743 biopsies	versus no rejection
		50 subAR	(CXCL9 AUROC 0.57; CXCL10 AUROC 0.64)
		243 no rejection	
[28]	Retrospective analysis, CXCL9 and CXCL10, Multicenter	373 biopsies	For subAR (excluding BL)
		45 subAR	versus no rejection
		283 no rejection	(multiparametric model including CXCL9 and CXLC10 AUROC 0.81)
[29]	Retrospective analysis, CXCL10, Single center	151 biopsies	For scTCMR versus no rejection (scABMR AUROC 0.80; scTCMR AUROC 0.78)
		23 scABMR	
		15 scTCMR	
		99 no ABMR	
		115 no TCMR	

AUROC, area under the ROC curve; subAR, subclinical rejection; BL, borderline rejection; scABMR, subclinical antibody-mediated rejection; scTCMR, subclinical; T cell-mediated rejection.

**TABLE 5:** Summary of literature review on urine chemokines for clinical acute rejection.

Authors	Study design	Number of samples	Results
[25]	Retrospective analysis, CXCL10, Single center	102 biopsies	For TCMR (cAR + subAR, excluding BL) vs. normal (AUROC 0.87)
		34 AR	
		22 normal	
[30]	Prospective analysis, CXCL9 and CXCL10, Multicenter	337 biopsies	For AR (cAR + subAR, excluding BL) versus no rejection
		45 AR	(CXCL9 AUROC 0.86; CXCL10 AUROC 0.77)
		228 no rejection	
[31]	Retrospective analysis, CXCL9 and CXCL10, Single center	281 biopsies	For clinical AR (excluding subAR and BL) versus no rejection
		78 AR	(CXCL9 AUROC 0.71; CXCL10 AUROC 0.76)
		203 no rejection	
[27]	Prospective analysis, CXCL9 and CXCL10, Single center	1722 samples	For clinical AR (excluding subAR and BL) versus no rejection
		743 biopsies	
		60 AR	(CXCL9 AUROC 0.72; CXCL10 AUROC 0.74)
		243 no rejection	
[28]	Retrospective analysis, CXCL9 and CXCL10, Multicenter	373 biopsies	For AR (cAR + subAR, excluding BL) vs. no rejection (multiparametric model including CXCL9 and CXLC10 AUROC 0.85)
		90 AR	
		283 no rejection	
[29]	Retrospective analysis, CXCL10, Single center	151 biopsies	For scTCMR versus normal (ABMR AUROC 0.76; TCMR AUROC 0.72)
		52 ABMR	
		36 TCMR	
		99 no ABMR	
		115 no TCMR	
[32]	Retrospective analysis, CXCL10, Single center	182 biopsies	For late clinical AR (excluding subAR and BL) versus normal (AUROC 0.72)
		55 AR	
		98 no rejection	

ABMR, antibody-mediated rejection; AR, acute rejection; AUROC, area under the ROC curve; BL, borderline rejection; cAR, clinical acute rejection; subAR, subclinical acute rejection; TCMR, T cell-mediated rejection.

Complete information including the list of consensus conference workgroup domains (and topics noted below), and process regarding consensus conference participant selection, development and refinement of consensus statements, and modified Delphi methodology including consensus polling, are previously reported in beforehand the in-person conference held in Prague, Czech Republic, 13–15 November 2022 [6].

### Overarching Statements From the Working Group

1. The majority of reviewed studies were conducted in adult patients; therefore, our recommendations are most applicable to the adult population. Our group acknowledged, however, that noninvasive biomarkers of rejection would be of great value in the care of pediatric kidney transplant recipients. Thus, we strongly encourage further study and development of these tests in the pediatric population. There are initial studies suggesting the potential utility of such monitoring in pediatric patients [34–36].
2. All of these diagnostic tests are not necessarily alloimmune-specific and thus, may be affected by sources of many other non-alloimmune inflammation such as infections and should be interpreted in that context.
3. Cost-benefit analyses were not considered in the forming of these statements but deserve further study.
4. All of these biomarker tests are available on more than one platform, but a paucity of head-to-head comparisons do not permit specific recommendations for one technique or specific test with a given technology (e.g., cell-free DNA) over another.
5. Most of these tests do not have validated cut-offs to interpret their output in a binary manner (high versus low-risk); therefore, the suggested threshold values should be taken with caution and their interpretation as a continuous variable may further help to translate the biological perturbation into a plausible clinical scenario.

## Results

### Donor-Derived Cell-Free DNA (dd-cfDNA)

Question 1 In kidney transplant patients with stable graft function, is plasma dd-cfDNA measurement a reliable diagnostic tool for subclinical acute rejection monitoring when compared with standard of care (eGFR/creatinine monitoring or surveillance biopsy)?

**Recommendation 1.1** - We suggest that clinicians consider measuring serial plasma dd-cfDNA in patients with stable graft function to exclude the presence of *subclinical* antibody-mediated rejection.
**Quality of Evidence** - Moderate
**Strength of Recommendation** - Weak in Favor

#### Comment to Recommendation 1.1

Concomitant testing for donor-specific HLA and non-HLA antibodies along with plasma dd-cfDNA may further increase the ability to detect the presence of antibody-mediated rejection (ABMR). Screening with dd-cfDNA alone does not appear to be a reliable tool for the detection of subclinical T-cell-mediated rejection (TCMR). Combining this test with other non-invasive biomarker technologies (gene expression profiling) may improve the detection of subclinical TCMR. The optimal timing and frequency of screening have not been established.

Question 2 In kidney transplant patients with acute allograft dysfunction, is plasma dd-cfDNA measurement a reliable diagnostic tool for acute rejection monitoring when compared with standard of care (eGFR/creatinine monitoring or for cause biopsy)?

**Recommendation 2.1** - We recommend that clinicians measure plasma dd-cfDNA in patients with acute graft dysfunction to exclude the presence of rejection, particularly antibody-mediated rejection.
**Quality of Evidence** - Moderate.
**Strength of Recommendation** – Moderate in Favor.

#### Comment to Recommendation 2.1

Concomitant testing for donor specific HLA and non-HLA antibodies along with plasma dd-cfDNA may further increase the ability to detect the presence of ABMR. Low levels of dd-cfDNA do not necessarily exclude the presence of TCMR in the graft.

#### Analytical Considerations Regarding dd-cfDNA

Currently, the donor-derived fraction of cell-free DNA is the standard measurement. Some groups have advocated for using both the fraction of dd-cfDNA and the total quantity of dd-cfDNA to improve the detection of clinical acute rejection.

Additionally, all dd-cfDNA assays in the US are currently being run in one of several central/reference labs (currently 3 commercially available assays that vary in the technique and number of single nucleotide polymorphisms analyzed). We recommend further studies to compare the available dd-cfDNA assays head-to-head to better define their performance compared to each other.

Different methodologies involving the assay being run in individual hospital labs used in Europe may require further validation for clinical correlation.

### Blood Gene Expression Profiling

Question 3 In kidney transplant patients with stable graft function, is blood gene expression profiling (GEP) a reliable diagnostic tool for subclinical acute rejection monitoring when compared with standard of care (eGFR/creatinine monitoring or surveillance biopsy)?

**Recommendation 3.1** - We do not yet recommend implementing the use of blood GEP to diagnose or exclude the presence of sub-clinical rejection.
**Quality of Evidence** – Low to Moderate.
**Strength of Recommendation** – Weak against.

#### Comment to Recommendation 3.1

Most of the published studies reviewed focused on using blood GEP in the setting of screening for subclinical rejection. Multiple GEP tests with differential performance were reviewed and detailed in Table 3. We strongly advocate the need to develop independent, prospective studies using GEP in stable patients to provide more robust evidence of its value to safely avoid surveillance biopsies.

Question 4 In kidney transplant patients with acute allograft dysfunction, is blood gene expression profiling (GEP) a reliable diagnostic tool for clinical acute rejection monitoring when compared with standard of care (eGFR/creatinine monitoring or for cause biopsy)?

**Recommendation 4.1** - We do not yet recommend the use of blood GEP to diagnose or exclude the presence of acute graft rejection in patients with acute allograft dysfunction.
**Quality of Evidence** – Low.
**Strength of Recommendation** – Weak against.

#### Comment to Recommendation 4.1

We strongly advocate the need to develop independent, prospective studies using GEP in the setting of graft dysfunction, to provide more robust evidence of its value to safely avoid or inform for-cause biopsies.

#### Analytical Considerations Regarding Gene Expression Profiling

Multiple research studies have investigated the value of blood GEP in stable patients to diagnose the presence of immune-mediated graft injury, regardless of the type of rejection. The aim of these biomarkers relies on trying to avoid unnecessary kidney allograft biopsies (for cause or for surveillance).

Blood GEP tests are all individual in their performance based on their initial derivation (cohort of patients, context of use), panel of specific genes, and locked classifier algorithm to interpret those genes. Therefore, different gene expression tests cannot be grouped together to analyze their performance.

Some studies have suggested that a combination of biomarkers (GEP with dd-cfDNA or functional cellular assays) may increase their predictive value [7], therefore such studies should be also considered and further validated.

### Urinary Chemokines

Question 5 In kidney transplant patients with stable allograft function, is urine chemokine measurement a reliable diagnostic tool for subclinical acute rejection monitoring when compared with standard of care (eGFR/creatinine monitoring or surveillance biopsy)?

**Recommendation 5.1** - We suggest the monitoring of a combination of urine CXCL9 and CXCL10 in stable patients to exclude subclinical rejection (TCMR or ABMR).
**Quality of Evidence** – Moderate.
**Strength of Recommendation** – Weak in Favor.

#### Comment to Recommendation 5.1

Use of this test in stable patients may help avoid the need for surveillance biopsies.

Question 6 In kidney transplant patients with acute allograft dysfunction, is urinary chemokine measurement a reliable diagnostic tool for clinical acute rejection monitoring when compared with standard of care (eGFR/creatinine monitoring or for-cause biopsy)?

**Recommendation 6.1** - We recommend the measurement of urinary chemokines CXCL9 and CXCL10 to inform the presence or absence of clinical acute rejection (TCMR or ABMR) in patients with graft dysfunction.
**Quality of Evidence** – Moderate.
**Strength of Recommendation** – Moderate in Favor.

#### Comment to Recommendation 6.1

None.

#### Analytical Considerations Regarding Urine Chemokine Profiling

Major strengths for urinary chemokine-based tests are the direct link between the biomarker and the underlying pathological mechanism, the reliance on multiple measurements in some longitudinal studies, and across different populations (American, European, Asian). Additionally, urinary chemokines are highly stable in urine samples.

Similar to dd-cfDNA platforms, some limitations for urinary chemokine-based predictions include the variable cutoffs according to different measurement techniques. We recommend further study to compare these tests across different platforms and to develop standardize thresholds.

A first randomized clinical trial by P. Hirt-Minkowski et al. investigating the clinical utility of renal allograft monitoring by urine CXCL10 chemokine was published in January 2023, after the Consensus Conference was held [37]. This study did not address the diagnostic performance of urinary CXCL10 to detect allograft rejection but if biopsies triggered by a limited number of urinary CXCL10 quantifications (at week-4, -10, -22) would impact on a composite endpoint at 1 year post-transplant (death-censored graft loss, clinical rejection between month 1 and 1 year, acute rejection in 1 year surveillance biopsy, chronic active T-cell–mediated rejection in 1 year surveillance biopsy, development of *de novo* donor-specific HLA antibodies, or eGFR <25 mL/min). In this landmark study, the primary composite endpoint was not met, underlining the need for further refinement in the methods and timing of posttransplant monitoring. However, the diagnostic performance of urinary CXCL10 to detect allograft rejection defined by the Banff 2019 classification was studied in an ancillary study and confirmed the diagnostic value of uCXCL10 (ROCAUC 0.73, *p* = 0.002). We believe that this study should provide a positive signal in the field, confirming the feasibility of implementing noninvasive biomarkers and prompting new interventional studies.

## Summary and Next Steps

The development and evolution of non-invasive molecular biomarkers of rejection in kidney transplant patients has started and will continue to revolutionize the care and management of patients. Here we provide a thorough review of the literature supporting these different molecular tests through mid-2022. Despite the number of published studies describing the diagnostic utility of these tests, the field still lacks from adequate perspective, interventional clinical trials demonstrating the value of using these biomarkers in prospective patient management.

## Data Availability

The original contributions presented in the study are included in the article/Supplementary Material, further inquiries can be directed to the corresponding author.
