# Supplementary material for: European Society of Organ Transplantation Consensus Statement on Testing for Non-Invasive Diagnosis of Kidney Allograft Rejection
Source: Transpl Int. 2024 Jan 4;36:12115. doi: 10.3389/ti.2023.12115 (PMC10794444; doi:10.3389/ti.2023.12115)
Supplement: Supplementary file 1 [file DataSheet1.docx]

**List of articles reviewed but not included in the main manuscript**

**Cell-free DNA**

1. Dauber EM, Kollmann D, Kozakowski N, et al. Quantitative PCR of INDELs to measure donor-derived cell-free DNA-a potential method to detect acute rejection in kidney transplantation: a pilot study. *Transplant International.* 2020;33(3):298-309.

2. Gielis EM, Ledeganck KJ, Dendooven A, et al. The use of plasma donor-derived, cell-free DNA to monitor acute rejection after kidney transplantation. *Nephrology, dialysis, transplantation : official publication of the European Dialysis and Transplant Association - European Renal Association.* 2020;35(4):714-721.

3. Melancon J. Donor Derived Cell Free DNA: is it all the same? *Kidney360.* 2020;1(10).

4. Zhang H, Zheng C, Li X, et al. Diagnostic Performance of Donor-Derived Plasma Cell-Free DNA Fraction for Antibody-Mediated Rejection in Post Renal Transplant Recipients: A Prospective Observational Study. *Frontiers in immunology.* 2020;11:342.

5. Cheng D, Liu F, Xie K, et al. Donor-derived cell-free DNA: An independent biomarker in kidney transplant patients with antibody-mediated rejection. *Transpl Immunol.* 2021:101404.

6. Bajaj A, Ihegword A, Qiu C, et al. Phenome-wide association analysis suggests the APOL1 linked disease spectrum primarily drives kidney-specific pathways. *Kidney international.* 2020;97(5):1032-1041.

7. Puliyanda DP, Swinford R, Pizzo H, Garrison J, De Golovine AM, Jordan SC. Donor-derived cell-free DNA (dd-cfDNA) for detection of allograft rejection in pediatric kidney transplants. *Pediatric transplantation.* 2021;25(2):e13850.

8. Qazi Y, Patel A, Fajardo M, et al. Incorporation of Donor-derived Cell-free DNA Into Clinical Practice for Renal Allograft Management. *Transplantation proceedings.* 2021;53(10):2866-2872.

9. Sawinski DL, Mehta S, Alhamad T, et al. Association between dd-cfDNA levels, de novo donor specific antibodies, and eGFR decline: An analysis of the DART cohort. *Clinical transplantation.* 2021;35(9):e14402.

10. Wolf-Doty TK, Mannon RB, Poggio ED, et al. Dynamic Response of Donor-Derived Cell-Free DNA Following Treatment of Acute Rejection in Kidney Allografts. *Kidney360.* 2021;2(4):729-736.

11. Butiu M, Obrisca B, Sibulesky L, et al. Donor-derived Cell-free DNA Complements De Novo Class II DSA in Detecting Late Alloimmune Injury Post Kidney Transplantation. *Transplantation direct.* 2022;8(2):e1285.

12. Guo L, Shen J, Lei W, et al. Plasma Donor-Derived Cell-Free DNA Levels Are Associated With the Inflammatory Burden and Macrophage Extracellular Trap Activity in Renal Allografts. *Frontiers in immunology.* 2022;13:796326.

13. Steggerda JA, Pizzo H, Garrison J, et al. Use of a donor-derived cell-free DNA assay to monitor treatment response in pediatric renal transplant recipients with allograft rejection. *Pediatric transplantation.* 2022;26(4):e14258.

14. Verhoeven J, Boer K, Peeters AMA, et al. A Novel High-Throughput Droplet Digital PCR-Based Indel Quantification Method for the Detection of Circulating Donor-derived Cell-free DNA After Kidney Transplantation. *Transplantation.* 2022;03:10.

**Gene Expression Profiling**

1. Peddi VR, Patel PS, Schieve C, Rose S, First MR. Serial Peripheral Blood Gene Expression Profiling to Assess Immune Quiescence in Kidney Transplant Recipients with Stable Renal Function. Ann Transplant [Internet]. 2020;25:e920839. Available from: https://ovidsp.ovid.com/ovidweb.cgi?T=JS&CSC=Y&NEWS=N&PAGE=fulltext&D=med17&AN=32341330

2. Marsh CL, Kurian SM, Rice JC, Whisenant TC, David J, Rose S, et al. Application of TruGraf v1: A Novel Molecular Biomarker for Managing Kidney Transplant Recipients With Stable Renal Function. Transplant Proc. 2019 Apr;51(3):722–8.

3. First MRR, Peddi VRR, Mannon R, Knight R, Marsh CLL, Kurian SMM, et al. Investigator Assessment of the Utility of the TruGraf Molecular Diagnostic Test in Clinical Practice. 2019 Apr;51(3):729–33. Available from: https://ovidsp.ovid.com/ovidweb.cgi?T=JS&CSC=Y&NEWS=N&PAGE=fulltext&D=med16&AN=30979457

4. Danger R, Paul C, Giral M, Lavault A, Foucher Y, Degauque N, et al. Expression of miR-142-5p in peripheral blood mononuclear cells from renal transplant patients with chronic antibody-mediated rejection. PLoS ONE [Electronic Resource] [Internet]. 2013;8(4):e60702. Available from: https://ovidsp.ovid.com/ovidweb.cgi?T=JS&CSC=Y&NEWS=N&PAGE=fulltext&D=med10&AN=23577151

5. Rascio F, Pontrelli P, Accetturo M, Oranger A, Gigante M, Castellano G, et al. A type I interferon signature characterizes chronic antibody-mediated rejection in kidney transplantation. Journal of Pathology [Internet]. 2015;237(1):72–84. Available from: https://ovidsp.ovid.com/ovidweb.cgi?T=JS&CSC=Y&NEWS=N&PAGE=fulltext&D=med12&AN=25925804

6. Celen E, Ertosun MG, Kocak H, Dinckan A, Yoldas B. Expression Profile of MicroRNA Biogenesis Components in Renal Transplant Patients. Transplant Proc [Internet]. 2017;49(3):472–6. Available from: https://ovidsp.ovid.com/ovidweb.cgi?T=JS&CSC=Y&NEWS=N&PAGE=fulltext&D=med14&AN=28340815

7. Zhang H, Huang E, Kahwaji J, Nast CC, Li P, Mirocha J, et al. Plasma Exosomes From HLA-Sensitized Kidney Transplant Recipients Contain mRNA Transcripts Which Predict Development of Antibody-Mediated Rejection. Transplantation [Internet]. 2017;101(10):2419–28. Available from: https://ovidsp.ovid.com/ovidweb.cgi?T=JS&CSC=Y&NEWS=N&PAGE=fulltext&D=med14&AN=28557957

8. Kurian SM, Velazquez E, Thompson R, Whisenant T, Rose S, Riley N, et al. Orthogonal Comparison of Molecular Signatures of Kidney Transplants With Subclinical and Clinical Acute Rejection: Equivalent Performance Is Agnostic to Both Technology and Platform. American Journal of Transplantation [Internet]. 2017;17(8):2103–16. Available from: https://ovidsp.ovid.com/ovidweb.cgi?T=JS&CSC=Y&NEWS=N&PAGE=fulltext&D=med14&AN=28188669

9. Pineda S, Sur S, Sigdel T, Nguyen M, Crespo E, Torija A, et al. Peripheral Blood RNA Sequencing Unravels a Differential Signature of Coding and Noncoding Genes by Types of Kidney Allograft Rejection. Kidney Int Rep. 2020 Oct;5(10):1706–21.

10. Shaw BI, Cheng DK, Acharya CR, Ettenger RB, Lyerly HK, Cheng Q, et al. An age-independent gene signature for monitoring acute rejection in kidney transplantation. Theranostics [Internet]. 2020;10(15):6977–86. Available from: https://ovidsp.ovid.com/ovidweb.cgi?T=JS&CSC=Y&NEWS=N&PAGE=fulltext&D=med17&AN=32550916

11. Mao YY, yang H, Wang M, Peng W, He Q, Shou ZF, et al. Feasibility of diagnosing renal allograft dysfunction by oligonucleotide array: Gene expression profile correlates with histopathology. Transpl Immunol [Internet]. 2011;24(3):172–80. Available from: https://ovidsp.ovid.com/ovidweb.cgi?T=JS&CSC=Y&NEWS=N&PAGE=fulltext&D=med8&AN=21130165

12. Van Loon E, Lerut E, de Loor H, Kuypers D, Emonds MP, Anglicheau D, et al. Antibody-mediated rejection with and without donor-specific anti-human leucocyte antigen antibodies: performance of the peripheral blood 8-gene expression assay. Nephrology Dialysis Transplantation. 2020 Aug 1;35(8):1328–37.

13. McDaniel DO, Rigney DA, McDaniel KY, Windham WJ, Redmond P, Williams B, et al. Early expression profile of inflammatory markers and kidney allograft status. Transplant Proc [Internet]. 2013 May [cited 2023 Mar 13];45(4):1520–3. Available from: https://pubmed.ncbi.nlm.nih.gov/23726610/

14. Ge YZ, Xu T, Cao WJ, Wu R, Yao WT, Zhou CC, et al. A Molecular Signature of Two Long Non-Coding RNAs in Peripheral Blood Predicts Acute Renal Allograft Rejection. Cell Physiol Biochem [Internet]. 2017 Nov 28 [cited 2023 Mar 13];44(3):1213–23. Available from: https://pubmed.ncbi.nlm.nih.gov/29179219/

15. Van Loon E, Lamarthee B, de Loor H, Van Craenenbroeck AH, Brouard S, Danger R, et al. Biological pathways and comparison with biopsy signals and cellular origin of peripheral blood transcriptomic profiles during kidney allograft pathology. Kidney Int [Internet]. 2022;102(1):183–95. Available from: https://ovidsp.ovid.com/ovidweb.cgi?T=JS&CSC=Y&NEWS=N&PAGE=fulltext&D=medl&AN=35526671

16. Crespo E, Roedder S, Sigdel T, Hsieh SC, Luque S, Cruzado JM, et al. Molecular and Functional Noninvasive Immune Monitoring in the ESCAPE Study for Prediction of Subclinical Renal Allograft Rejection. Transplantation [Internet]. 2017 [cited 2023 Mar 13];101(6):1400–9. Available from: https://pubmed.ncbi.nlm.nih.gov/27362314/

17. Cheung R, Xu H, Jin X, Tian W, Pinney K, Bu L, et al. Validation of a gene expression signature to measure immune quiescence in kidney transplant recipients in the CLIA setting. Biomark Med [Internet]. 2022 Jun 1 [cited 2023 Mar 13];16(8):647–61. Available from: https://pubmed.ncbi.nlm.nih.gov/35485169/

**Urine chemokines**

The list of articles which were reviewed but not presented in the main manuscript.

1. Ho J, Rush DN, Krokhin O, et al. Elevated Urinary Matrix Metalloproteinase-7 Detects Underlying Renal Allograft Inflammation and Injury. *Transplantation*. 2016; **100**: 648-54.

2. Ho J, Schaub S, Wiebe C, et al. Urinary CXCL10 Chemokine Is Associated With Alloimmune and Virus Compartment-Specific Renal Allograft Inflammation. *Transplantation*. 2018; **102**: 521-9.

3. Muhlbacher J, Doberer K, Kozakowski N, et al. Non-invasive Chemokine Detection: Improved Prediction of Antibody-Mediated Rejection in Donor-Specific Antibody-Positive Renal Allograft Recipients. *Front Med (Lausanne)*. 2020; **7**: 114.

4. Gielis EM, Anholts JDH, van Beelen E, et al. A Combined microRNA and Chemokine Profile in Urine to Identify Rejection After Kidney Transplantation. *Transplantation Direct*. 2021; **7**: e711.

5. Hirt-Minkowski P, Amico P, Ho J, et al. Detection of clinical and subclinical tubulo-interstitial inflammation by the urinary CXCL10 chemokine in a real-life setting. *Am J Transplant*. 2012; **12**: 1811-23.

6. Ho J, Sharma A, Mandal R, et al. Detecting Renal Allograft Inflammation Using Quantitative Urine Metabolomics and CXCL10. *Transplant Direct*. 2016; **2**: e78.

7. Jackson JA, Kim EJ, Begley B, et al. Urinary chemokines CXCL9 and CXCL10 are noninvasive markers of renal allograft rejection and BK viral infection. *American Journal of Transplantation*. 2011; **11**: 2228-34.

8. Raza A, Firasat S, Khaliq S, et al. The association of urinary interferon-gamma inducible protein-10 (IP10/CXCL10) levels with kidney allograft rejection. *Inflamm Res*. 2017; **66**: 425-32.

9. Seiler LK, Phung NL, Nikolin C, et al. An Antibody-Aptamer-Hybrid Lateral Flow Assay for Detection of CXCL9 in Antibody-Mediated Rejection after Kidney Transplantation. *Diagnostics (Basel)*. 2022; **12**: 25.
